# Introduced *Drosophila subobscura* populations perform better than native populations during an oviposition choice task due to increased fecundity but similar learning ability

**DOI:** 10.1002/ece3.2015

**Published:** 2016-02-16

**Authors:** Julien Foucaud, Céline Moreno, Marta Pascual, Enrico L. Rezende, Luis E. Castañeda, Patricia Gibert, Frederic Mery

**Affiliations:** ^1^ Laboratoire Evolution, Génomes, Comportement et EcologieUMR‐CNRS 9191Gif/YvetteFrance; ^2^ Department of Genetics and IrBioUniversitat de BarcelonaAv. Diagonal 64308028BarcelonaSpain; ^3^ Department of Life SciencesUniversity of RoehamptonHolybourne AvenueLondonSW15 4JDUK; ^4^ Instituto de Ciencias Ambientales y EvolutivasUniversidad Austral de ChilePO 5090000ValdiviaChile; ^5^ Université de LyonUniversité Lyon1Laboratoire de Biométrie et Biologie EvolutiveUMR CNRS 555843 Bd du 11 Novembre 191869622Villeurbanne CedexFrance; ^6^ UMR 1062 CBGP (INRA, IRD, CIRAD, Montpellier SupAgro)INRA755 Avenue du campus Agropolis34988Montferrier/LezFrance

**Keywords:** Biological invasion, *Drosophila*, learning, phenotypic plasticity, trade‐off

## Abstract

The success of invasive species is tightly linked to their fitness in a putatively novel environment. While quantitative components of fitness have been studied extensively in the context of invasive species, fewer studies have looked at qualitative components of fitness, such as behavioral plasticity, and their interaction with quantitative components, despite intuitive benefits over the course of an invasion. In particular, learning is a form of behavioral plasticity that makes it possible to finely tune behavior according to environmental conditions. Learning can be crucial for survival and reproduction of introduced organisms in novel areas, for example, for detecting new predators, or finding mates or oviposition sites. Here we explored how oviposition performance evolved in relation to both fecundity and learning during an invasion, using native and introduced *Drosophila subobscura* populations performing an ecologically relevant task. Our results indicated that, under comparable conditions, invasive populations performed better during our oviposition task than did native populations. This was because invasive populations had higher fecundity, together with similar cognitive performance when compared to native populations, and that there was no interaction between learning and fecundity. Unexpectedly, our study did not reveal an allocation trade‐off (i.e., a negative relationship) between learning and fecundity. On the contrary, the pattern we observed was more consistent with an acquisition trade‐off, meaning that fecundity could be limited by availability of resources, unlike cognitive ability. This pattern might be the consequence of escaping natural enemies and/or competitors during the introduction. The apparent lack of evolution of learning may indicate that the introduced population did not face novel cognitive challenges in the new environment (i.e., cognitive “pre‐adaptation”). Alternatively, the evolution of learning may have been transient and therefore not detected.

## Introduction

Biological invasions pose a serious threat to both natural ecosystems and human health and economy (Mooney and Hobbs 2000). A detailed understanding of the process of biological invasions and of the characteristics of invasive species is required to predict and manage invasion risks. A long‐standing goal of invasion biology is thus to characterize life history traits that enable particular species, populations or individuals to become successful invaders, that is, to overcome geographic, ecological and/or demographic barriers to transport, establishment, and spread (Sakai et al. 2001; Blackburn et al. 2011; Chapple et al. 2012).

During the establishment phase, introduced individuals may face novel environmental conditions, for example, new climatic conditions, competitors, predators, parasites, or food sources. They may also face new demographic challenges, such as reduced mate density. An introduced species' success is directly related to its fitness in its new habitat, which is likely to depend both on phenotypic plasticity (Davidson et al. 2011; Lande 2015) and genetic change (Dybdahl and Kane 2005; Dlugosch and Parker 2008; Colautti and Lau 2015). The genetic basis for increased fitness of an introduced species in a novel environment could simply come from random drift, with the initial colonization involving a particular subset of migrants with beneficial pre‐adaptations. Alternatively, it could come from local adaptation after a few generations that may or may not include admixture of previously isolated genotypes (Huey et al. 2000; Lee 2002; Barker et al. 2009; Geiger et al. 2011; Moran and Alexander 2014). In any case, heritable fitness‐related traits are expected to evolve rapidly after introduction to a novel environment (Simões et al. 2008; Santos et al. 2012), because traits closely associated with fitness should rapidly fix the alleles responsible for higher fitness (Merilä and Sheldon 2000).

In most invertebrates, fitness depends strongly on oviposition (Doak et al. 2006 but see Fincke and Hadrys 2001), which can be broken down into a fecundity component (Sgró and Hoffmann 1998; Long et al. 2009) (the number of eggs that are actually laid) and a behavioral component (the ability to detect and choose suitable oviposition sites (Thompson and Pellmyr 1991; Papaj and Prokopy 1989; Egas and Sabelis 2001). The contribution of fecundity to fitness can be measured quantitatively as the raw number of eggs laid. The contribution of oviposition choice behavior to fitness is more qualitative (i.e., different oviposition site choices will result in offspring of different quality), even if it is also quantitatively measured (e.g., as number of eggs laid in good vs. bad oviposition sites). An introduced species is more likely to establish in a new environment if it has both high fecundity and behavioral plasticity. However, both can also carry fitness‐related costs (Reznick 1985; Chippindale et al. 1993; Reznick et al. 2000; Mery and Kawecki 2003, 2004). It is unclear whether individuals that successfully establish in novel environments allocate more to one or both components, or to neither. Some studies – primarily in plants and often as part of the “Evolution of Increased Competitive Ability” framework (EICA; Blossey and Notzold 1995; Meyer et al. 2005) – have looked at the evolution of fecundity. They found that fecundity has played a pivotal role in some invasion events (Leger and Rice 2003; Meyer and Hull‐Sanders 2008; Horkova and Kovac 2014). Other comparative studies in birds (Sol et al. 2002, 2005), mammals (Sol et al. 2008), amphibians, and reptiles (Amiel et al. 2011) have shown that invasive species often have a high relative brain size and a high foraging innovation frequency. However, larger brains do not necessarily translate into higher cognitive abilities (Bezzina et al. 2014; Roth et al. 2010a; but see Kotrschal et al. 2014). The few studies that have explored the interaction between fecundity and the capacity to learn show a negative correlation between them (Mery and Kawecki 2004; Snell‐Rood et al. 2011). To date, no studies have investigated variation both in fecundity and behavioral plasticity in the context of a biological invasion.

In the present study, we directly tested putative differences in oviposition performance between introduced and native populations of an invasive species, and whether they rely on learning (“quality” of oviposition) and/or fecundity (“quantity” of oviposition). We used *Drosophila subobscura* as a model species. *Drosophila* species are known to display learning and memory in a variety of tasks, such as those related to mate choice, choice of food source, and spatial localization of preferred sites using a variety of environmental cues (olfactory, gustatory, visual, social) (Mery and Kawecki 2002; Dukas 2005; Kawecki 2010; Battesti et al. 2012; Foucaud et al. 2013). *D. subobscura* is a native European species that has successfully invaded South and North America from a reduced number of colonizers (Pascual et al. 2007). In particular, there is a well‐documented, ongoing invasion on the coast of Chile that began with the introduction of *D. subobscura* to Puerto Montt in the late 1970s showing quick evolutionary responses in several adaptive traits such as wing size (Brncic et al. 1981; Balanya et al. 1994; Gilchrist et al. 2004; Fernandez Iriarte et al. 2009). We tested for intraspecific differences in oviposition using an ecologically relevant cognitive task that assessed both fecundity and behavioral plasticity. We analyzed whether either of the two components of fitness differed between native and invasive populations.

## Materials and Methods

### Sampling

**Figure 1:**
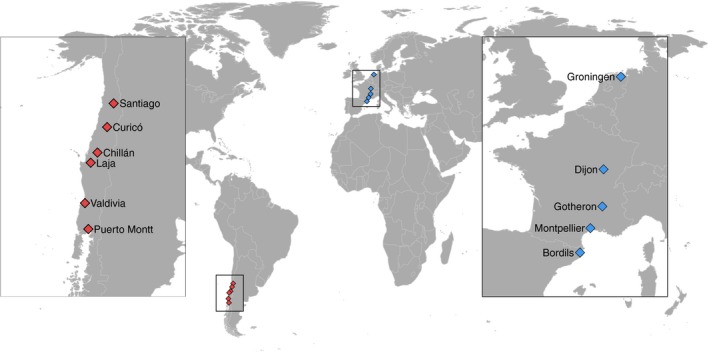
Map of the sampled native (blue) and invasive (red) *D. subobscura* populations.

Several hundred flies were collected both in the native (Europe) and introduced (South America) ranges of *D. subobscura* in 2009 and 2010. In the native range, five populations were collected along a north–south transect from the Netherlands to Spain (Table 1, Fig. 1). In the introduced range, six populations were collected along a similar south–north transect from Puerto Montt (the original introduction site) to Santiago (Table 1, Fig. 1). Sampled populations from both the introduced and native ranges were also used in several other studies (Rezende et al. 2010; Calabria et al. 2012; Simões et al. 2012; Castañeda et al. 2013). We collected flies in public urban areas that did not require authorization for sampling. The only exception was the French Gotheron sample, for which we had authorization to sample from a public science institute's orchard.

**Table 1:** Sampling design for native and introduced *D. subobscura* populations

Range	Country	Site	Latitude	Longitude	Sampling date
Native	Netherlands	Groningen	53°12′5″N	6°34′36″E	August 2009
Native	France	Dijon	47°17′47″N	5°2′26″E	August 2009
Native	France	Gotheron	44°55′23″N	4°55′50″E	March 2010
Native	France	Montpellier	43°36′10″N	3°51′20″E	September 2009
Native	Spain	Bordils	42°1′29″N	2°54′54″E	April 2010
Introduced	Chile	Santiago	33°30′16″S	70°39′37″W	October 2010
Introduced	Chile	Curico	34°59′42″S	71°14′47″W	October 2010
Introduced	Chile	Chillan	36°36′47″S	72°6′26″W	October 2010
Introduced	Chile	Laja	37°16′35″S	72°42′42″W	October 2010
Introduced	Chile	Valdivia	39°50′3″S	73°13′19″W	October 2010
Introduced	Chile	Puerto Mont	41°28′22″S	72°57′44″W	October 2010

To avoid maternal effects, all flies were kept in the laboratory for at least two generations before starting the experimental protocol. There were no systematic differences in how long native and introduced flies were maintained in, and therefore able to adapt to, the laboratory. Fly stocks were maintained in the laboratory in 50‐mL tubes (around 100 flies per tube) on standard food medium at 21 °C on 14 h/10 h light/dark cycles. To test for oviposition site learning, we simultaneously collected males and females in batches of approximately 30 individuals, upon emergence. We kept them together for 7 days and separated females from males on ice 6 h before the start of the experiment (Battesti et al. 2012). The use of 7‐day‐old mated females was selected because it corresponds to the maximum sexual activity of *D. subobscura* flies (Pascual et al. 1990) and is considered the upper period for studies on early fecundity, showing quick and significant response in laboratory foundations (Simões et al. 2008; Santos et al. 2012).

### Oviposition site learning experiment

To test for differences in oviposition between native and invasive populations, while also decomposing learning and fecundity components, we trained female flies to associate an aversive gustatory cue to a particular odor on an oviposition site. Our learning protocol was divided into a conditioning phase and a test phase.

The conditioning phase lasted 12 h. Groups of 12 females were placed into a 120 × 50 × 90 mm plastic cage in constant light and were given the choice between two oviposition substrates: one flavored with a banana odor and the other with a strawberry odor. These odors were chosen because (1) they were commercially available natural compounds that allow relatively high level of replication (contrary to homemade extracts or juices), (2) they relate to food sources that are present in both ranges (i.e., strawberry is naturally present and banana is imported in both Chile and Europe). Preliminary experiments showed that flies were attracted by these odorants. One of the two media was supplemented with 3 g/L of quinine, an aversive gustatory compound. The oviposition substrate was a mixture of 20 g/L sucrose, 10 g/L agar, and 6 mL/L of artificial banana or strawberry odor (Gazignaire SA, La Roquette‐sur‐Siagne, France) poured into a 35‐mm Petri dish (Battesti et al. 2012). Pilot experiments showed that in the absence of quinine and at this odor concentration all natural *D. subobscura* populations tested laid eggs on both oviposition sites without preference for either medium. In half of the replicates, quinine was added into the banana‐flavored substrate, and in the other half, it was added to the strawberry‐flavored substrate. Thus, for each experiment, half of the female groups were trained to avoid banana and the other half were trained to avoid strawberry. To test whether females use past experience to modify their preference, flies were tested immediately after training.

Our test phase was divided into two sections. In the first test phase (0–8 h), both oviposition media were replaced with fresh, quinine‐free media. Flies were then allowed to lay eggs for 8 h without interruption. In the second test phase (8–20 h), oviposition media were again replaced with fresh media, and flies were allowed to lay eggs for 12 additional hours. Observers that were blind with respect to the treatment counted the number of eggs laid on each medium.

For each population, we simultaneously tested eight replicate groups of 12 flies on each conditioning substrate (banana or strawberry associated with quinine) for a total of 16 test boxes per population. All 11 populations were tested simultaneously constituting an experimental block of 176 boxes. We then replicated the previous experimental block six times, for a total of 1056 test boxes (see Table S1 for details). Females laid eggs in 1042 of the 1056 test boxes (98.7%). We discarded test boxes without eggs (1.3%) from the analysis. In total, 108,098 eggs were counted on the oviposition substrates during the test phases. All tests were performed in a temperature‐controlled chamber set at 21 °C. All datasets are available from the Dryad Digital Repository: http://dx.doi.org/10.5061/dryad.d8d01.

### Data analysis

In this experimental protocol, oviposition performance was measured as the total number of eggs laid on the correct medium (banana for flies conditioned to avoid strawberry, and strawberry for flies conditioned to avoid banana). Thus, oviposition performance depends on both fecundity and learning ability. We used the proportion of eggs laid in the “correct” substrate to compare learning ability between populations from different ranges. We also measured the fecundity of females from different ranges as the total number of eggs laid through the experiment on any substrate.

Number of observations was both large and only slightly unbalanced (Table S1). For all variables, we performed a generalized linear mixed model (GLMM) analysis with Laplace approximation (Bolker et al. 2009), using the lme4 package (Bates et al. 2014) in the R statistical framework (R Core Team, 2008). For the proportion of eggs laid in the correct substrate (proportion data), we ran a binomial GLMM with the logit link function. For both the number of eggs laid in the correct substrate and the total fecundity (count data), we ran a Poisson GLMM with a log link. For all variables, we investigated the significance of the same explanatory factors: “type” (the type of range: native or invasive), “pop” (the population sampled, nested in type), “test” (the test phase: 0–8 h or 8–20 h), “cond” (conditioned to avoid strawberry or banana), and “date” (the date the experiment took place). Both “pop” and “date” were treated as random effects, while “type,” “test,” and “cond” were treated as fixed effects. To account for overdispersion in our variables, we added an observation‐level random effect to the models as recommended by Bolker et al. (Bolker et al. 2009). We followed a step‐by‐step procedure of model simplification from the full model (starting with random effects and then fixed effects) based on the Akaike information criterion, and tested the significance of the remaining effects via likelihood ratio tests (LRT). We calculated adjusted means and confidence intervals for each significant fixed factor using a bootstrap resampling procedure (1000 resampling of 500 observations each) with the boot package (Canty and Ripley 2015). For all variables (number and proportion of correct oviposition choices and total fecundity), our main goal was to test the effect of the type of population (native vs. invasive). We tested if learning occurred in native and invasive populations of *D. subobscura* using binomial tests for each “type” × “test” combination using the proportion of eggs laid in the correct substrate. A proportion significantly higher than 0.5 indicated that flies had learned.

To test for a putative interaction between fecundity and learning performance that might differ between native and invasive populations, we performed Pearson's product‐moment correlation tests for each “type” × “test” combination (factors that significantly influenced fecundity; see “Results”). A significant negative correlation would suggest that the total number of eggs laid modifies the proportion of eggs laid on the correct medium or that learning affects fecundity (probably through energy allocation). Significant differences in “type” correlation coefficients would suggest that fecundity differentially alters the learning output signal or that learning differentially affects fecundity in native and invasive populations.

## Results

### Does invasive *D. subobscura* lay more eggs on the correct medium than native individuals?

**Figure 2:**
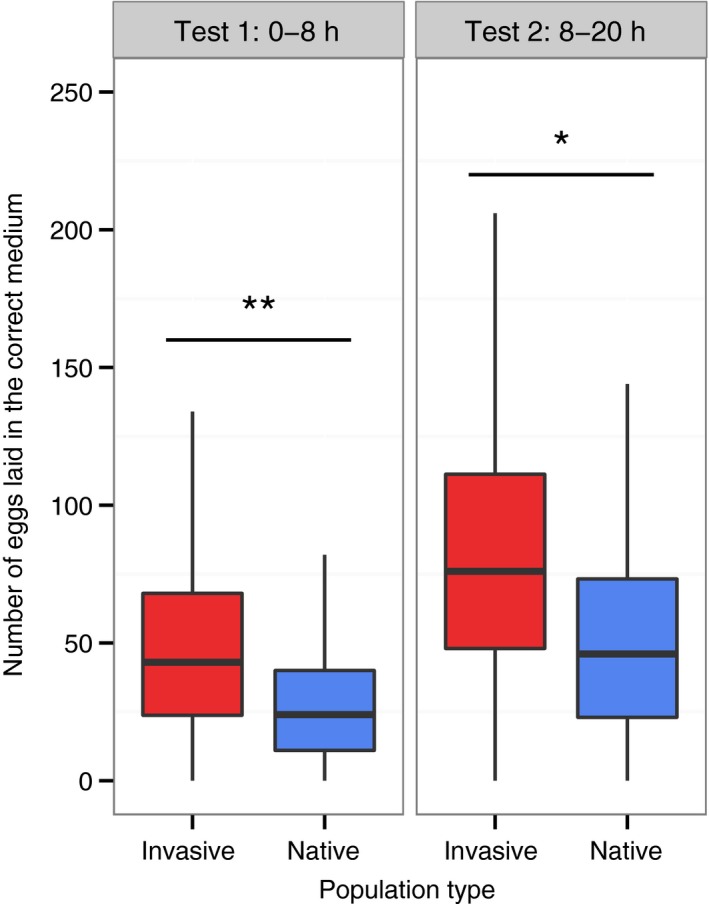
Boxplot of the number of eggs that native (blue) and invasive (red) *D. subobscura* females laid in the correct medium during test phase 1 (0–8 h) and test phase 2 (8–20 h). Females from invasive populations had higher fecundity both during the test phase 1 (LRT: χ_1_ = 9.190, *P* = 0.002) and test phase 2 (LRT:* χ*_1_ = 6.369, *P* = 0.012). **P < 0.01, *P < 0.05.

Our results showed that invasive populations laid significantly more eggs on the correct medium over the course of the experiment than did the native females (Fig. 2; Adjusted means [lower; upper 95% confidence interval (CI)]: *n*_correct eggs_ (native) = 39.44 [36.66; 42.20], *n*_correct eggs_ (invasive) = 71.69 [66.78; 76.77]; LRT: $\chi_{1}^{2}$ = 9.884, *P* = 0.002). Significantly more eggs were laid on the correct medium during the second test phase (“test” effect: *n*_correct eggs_ (test 1) = 44.16 [40.93; 47.69], *n*_correct eggs_ (test2) = 80.83 [75.54; 85.93]; LRT: $\chi_{1}^{2}$ = 10.02, *P* = 0.002), as expected from its longer duration (12 h vs. 8 h). When using a time “adjusted” dataset with a second test phase of 8 h (assuming a linear relationship between time and egg‐laying), similar number eggs were laid in the correct medium during both phases (adjusted “test” effect: *n*_correct eggs_ (test 1) = 42.14 [38.92; 45.11], *n*_correct eggs_ (test2) = 50.44 [47.65; 53.60]; LRT: $\chi_{1}^{2}$ = 2.53, *P* = 0.11). It is noteworthy that the conditioning led to a significantly greater “correct” output when flies were conditioned to avoid banana than when conditioned to avoid strawberry medium (“cond” effect: *n*_correct eggs_ (avoid banana) = 68.69 [61.93; 76.24], *n*_correct eggs_ (avoid strawberry)  = 44.93 [41.09; 49]; LRT: $\chi_{1}^{2}$ = 10.301, *P* = 0.001). We found no significant interaction between fixed factors (type of population, test phase, and conditioning odor; all *P* > 0.2).

### Do native and invasive *D. subobscura* differ in their ability to learn correct oviposition sites?

**Figure 3:**
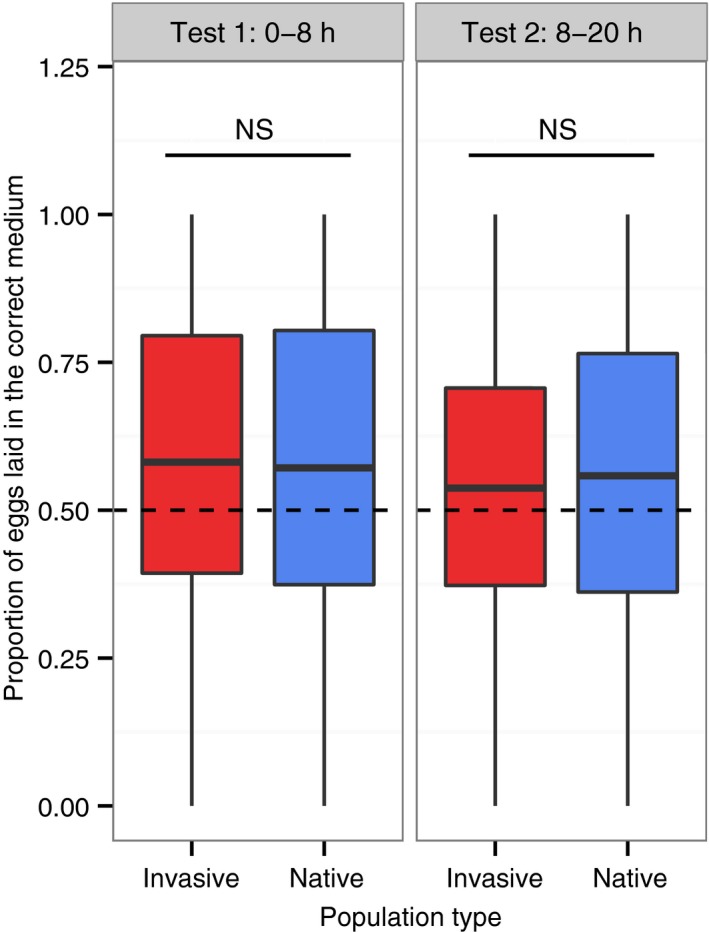
Boxplot of the proportion of eggs laid in the correct medium of native (blue) and invasive (red) *D. subobscura* females in the oviposition site learning assay during test phase 1 (0–8 h) and test phase 2 (8–20 h). NS:* P* > 0.05; n = 1042 tests (see Table S1 for details); NS: *P* < 0.05.

Both native and invasive populations laid significantly more than 50% of their eggs on the correct substrate in the first test phase (95% CI for native populations = [0.549–0.566], *P* < 0.001; 95% CI for invasive populations = [0.568–0.580], *P* < 0.001), indicating that flies learned in response to conditioning (Fig. 3). Similar results were found during the second test phase, both for native (95% CI = [0.532–0.545], *P* < 0.001) and invasive populations (95% CI = [0.531–0.541], *P* < 0.001).

Native and invasive populations did not differ in their ability to respond to the conditioning procedure. Our GLMM analysis indicated no effect of a population's range on the proportion of eggs laid in the correct substrate (“type” effect, LRT: $\chi_{1}^{2}$ = 0.163, *P* = 0.686). This result held no matter how much time had passed since conditioning (no “type” × “test” interaction; LRT: $\chi_{1}^{2}$ = 0.164, *P* = 0.685). In contrast, time since conditioning significantly affected learning ability (“test” effect LRT: $\chi_{1}^{2}$ = 10.908, *P* = 0.001)—learning decayed during the second test phase. Additionally, the type of conditioning (whether to avoid banana or strawberry) had a significant effect on the proportion of eggs laid on the correct medium (“cond” effect LRT: $\chi_{1}^{2}$ = 345.95, *P* < 0.001)—flies learned better when trained to avoid banana and lay on strawberry media.

### Do invasive *D. subobscura* populations have higher fecundity than native populations?

**Figure 4:**
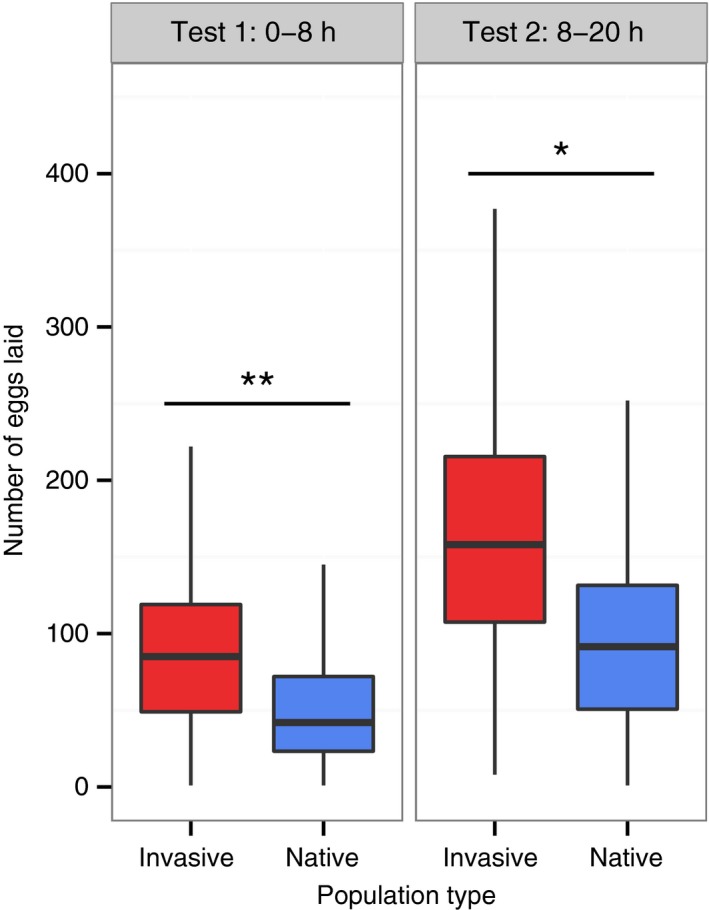
Boxplot of the total fecundity (in number of eggs laid) of native (blue) and invasive (red) *D. subobscura* females during test phase 1 (0–8 h) and test phase 2 (8–20 h). Females from introduced populations had a higher fecundity both during test 1 (LRT:* χ*_1_ = 8.197, *P* = 0.004) and test 2 phases (LRT:* χ*_1_ = 6.369, *P* = 0.011). **P < 0.01, *P < 0.05.

Native and invasive populations showed marked differences in total fecundity. Our results demonstrated that females from invasive populations laid more eggs than females from native populations (Fig. 4; “type” effect: *n*_total eggs_ (native) = 68.82 [65.17; 72.57], *n*_total eggs_ (invasive) = 126.47 [119.70; 133.91]; LRT: $\chi_{1}^{2}$ = 9.122, *P* = 0.003). This result held no matter how much time had passed after conditioning (no “type” × “test” interaction; LRT: $\chi_{1}^{2}$ = 0.021, *P* = 0.886). All females laid more eggs during the 12 h long, second test phase than during the 8 h long, first test phase (Fig. 4; “test” effect: *n*_total eggs_ (test 1) = 70.72 [66.40; 74.97], *n*_total eggs_ (test 2) = 140.87 [134.46; 148.09]; LRT: $\chi_{1}^{2}$ = 10.583, *P* = 0.001). Adjusting for the longer time of the second test phase tended to remove this difference (adjusted “test” effect: LRT: $\chi_{1}^{2}$ = 3.43, *P* = 0.064). Fecundity was slightly higher in females from both native and invasive populations when first conditioned to avoid strawberry (“cond” effect: *n*_total eggs_ (avoid banana) = 98.81 [89.92; 107.24], *n*_total eggs_ (avoid strawberry) = 108.60 [99.82; 117.40]; LRT: $\chi_{1}^{2}$ = 3.406, *P* = 0.065). Overall, whatever the correct odor used and timing of test, females from invasive populations always laid more eggs than females from native populations.

### Does fecundity interact with learning of oviposition site?

**Figure 5:**
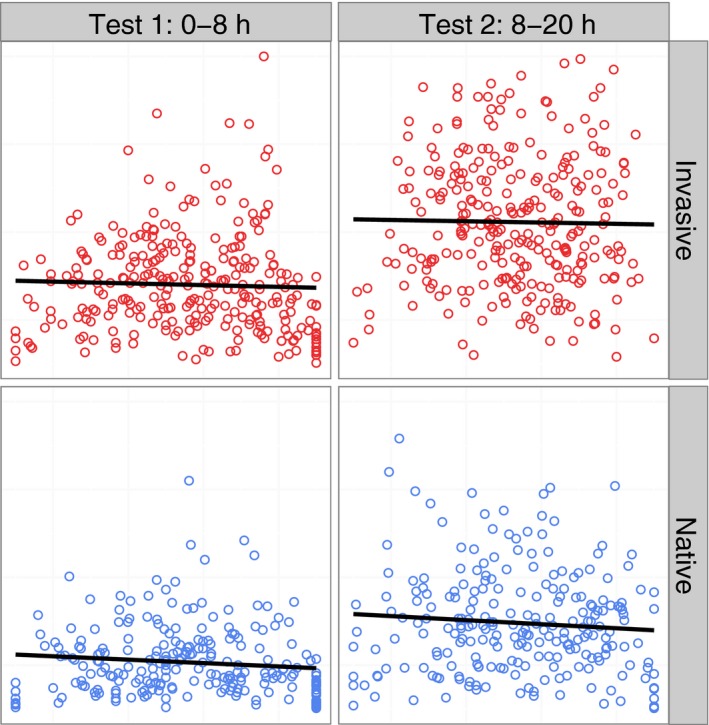
Correlation between fecundity and learning in native (blue) and invasive (red) *D. subobscura* females during test phase 1 (0–8 h) and test phase 2 (8–20 h) phases. Fecundity is expressed in total number of eggs laid, and learning is measured through the proportion of eggs laid in the correct medium. *n* = 1042 tests. Black segments represent linear regressions for each type of population and test phase combination.

Native and invasive populations did not show any interaction between fecundity and learning ability. Indeed, all correlation coefficients tested were close to zero and not significant (Fig. 5; Pearson's product‐moment correlation tests: native × test 1: *ρ *= −0.107, *t*_232_ = −1.64, *P* = 0.102; native × test 2: *ρ* = −0.081, *t*_234_ = −1.25, *P* =0.214; invasive × test 1: *ρ *= −0.037, *t*_283_ = −0.622, *P* = 0.534; invasive × test 2: *ρ *= −0.029, *t*_285_ = −0.49, *P* = 0.625). Our results unambiguously demonstrate that the proportion of eggs laid on the correct oviposition site was not significantly linked to fecundity.

## Discussion

In evolutionary biology, it is recognized that both phenotypic plasticity and genetic change are possible (and nonexclusive) pathways to successfully deal with novelty and changing environments. In an invasion biology context, phenotypic plasticity might facilitate survival and reproduction during the establishment phase of invasive species, where novel biotic and abiotic conditions may occur (Davidson et al. 2011). On the other hand, random drift (i.e., founder effect), admixture and/or adaptation to local conditions can result in genetic change promoting the establishment and spread of introduced populations (Maron et al. 2004; Blumenthal and Hufbauer 2007; Lavergne and Molofsky 2007; Alexander et al. 2009; Ebeling et al. 2011). Our study focused on putative change in oviposition performance in an invasive invertebrate model species and asked whether it relies on changes in fecundity and/or behavioral plasticity.

Our results showed that oviposition performance was higher in invasive than in native populations. Indeed, invasive females consistently laid more eggs in the correct oviposition medium (our proxy for fitness; see below) than native females. Both native and invasive *D. subobscura* were equally able to learn the relative qualitative value of two oviposition sites and express their learned preference through their choice of oviposition site. As a corollary to these results, we did not detect any interaction between fecundity and learning during this task in *D. subobscura* and, in particular, no trade‐off. One could argue that differences in oviposition performance between invasive and native females might be because they differed in how each odor used in our protocol stimulated oviposition (e.g., invasive females could have been more stimulated by the scent than were native females). However, our results showed that although both had a slight preference for the strawberry odor, invasive females outperformed native females no matter what was the “correct” odor (banana or strawberry) and no matter how much time had passed since conditioning (test phases 1 and 2). These results strongly suggest that oviposition performance is generally higher in invasive females than in native females. Additionally, both odors have been used routinely in learning paradigms without stimulating or preventing oviposition in a wide variety of *Drosophila* species (e.g., *D. melanogaster*,* D. simulans*,* D. seychellia*,* D. mauritiana*; F. Mery and J. Foucaud, unpubl. results). In the context of invasion biology, these odors have no reason to be differently used as a cue for oviposition in a given range (i.e., no fruit is present in one range and absent in the other).

The findings of our study are consistent with recent evidence that invasive populations have higher fitness than their counterparts in their native range. Invaders may benefit from a variety of novel ecological or genetic conditions: escape from co‐evolved enemies (i.e., the Enemy‐Release Hypothesis, ERH; Maron and Vila 2001; Keane and Crawley 2002; Colautti et al. 2004) or competitors (EICA; Blossey and Notzold 1995; Meyer et al. 2005), and/or improved genetic variance (e.g., from multiple introductions; (Durka et al. 2005; Lavergne and Molofsky 2007; Kajita et al. 2012). In the particular case of *D. subobscura*, we must note that (1) the South American invasion originated from a single introduction of fewer than 12 individuals (Pascual et al. 2007), limiting initial genetic diversity (i.e., reduced genetic variance, at least at neutral markers), and (2) *D. subobscura* is notorious for being a poor competitor (Budnik et al. 1997; Pascual et al. 1998) that may have benefitted from an escape from competitors when introduced in Chile (Budnik et al. 1997). Our study helps elucidate the relative contributions of fecundity and behavioral plasticity in the emergence of this invasive phenotype.

Because both improved fecundity and improved learning abilities entail some costs (Reznick 1985; Chippindale et al. 1993; Reznick et al. 2000; Mery and Kawecki 2003, 2004), there was no clear expectation of a particular direction of change in either trait due to introduction in a putatively novel habitat. However, we expected a trade‐off between the use of learning and fecundity, based on previous studies (Mery and Kawecki 2004; Snell‐Rood et al. 2011). Our results demonstrated that the improved performance of invasive populations was limited to an increase in the quantitative component of oviposition (fecundity), with no change in the qualitative component (learning ability). This result might indicate an acquisition trade‐off rather than allocation trade‐off between populations from different ranges (Reznick et al. 2000) (i.e., invasive genotypes putatively better than native ones in acquiring resources, thus enabling them to pay off the cost of improved fecundity). This acquisition trade‐off might be mediated by an escape from co‐evolved enemies. Alternatively, our results could be explained by an allocation trade‐off between reproduction and an unknown trait, independent from learning. These hypotheses still need to be directly tested. The lack of negative correlation between fecundity and learning ability may indicate that the benefits of learning were not counter‐balanced by the cost of increased egg production in the correct medium. Additionally, an artificial increase in mating activity (Pascual et al. 1990) and early fecundity (Santos et al. 2012) has been observed over time in laboratory‐reared populations of *D. subobscura*. However, in our study, some of the less fecund native populations had been sampled earlier and laboratory‐reared for a longer period than had the more fecund invasive populations (Table 1). Our observations are thus conservative and our conclusion remains valid when taking putative postsampling laboratory evolution into account.

Two alternative hypotheses could explain why the learning ability of invasive populations of *D. subobscura* did not increase beyond that of the native populations. First, there is an a priori assumption that the introduced environment is fundamentally different from the native one and is a significant selective barrier. But this assumption is rarely tested in invasion biology and may not be valid. Invasive species like *D. subobscura* that are found in human‐modified environments in their native range are more likely to face similar environmental conditions in the introduced range, given the homogenizing nature of the global anthropization of ecosystems (McKinney and Lockwood 1999; McKinney 2006). Furthermore, as invasive species are usually transported via human activities from one hub to another, environmental conditions are likely to be similar in the native and introduced ranges (Tatem and Hay 2007). In the case of *D. subobscura*, native habitats comprise urban areas that may select for higher learning ability (Lowry et al. 2013), suggesting that introduced individuals may be adequately “preadapted” (Prevosti et al. 1988). It is thus plausible that cognitive challenges are similar in both *D. subobscura*'s introduced and native ranges. This highlights the need to perform careful ecological studies before cognitive tests (e.g., studies of the ecological gradient in cognitive ability found in the black‐capped chickadees (Roth and Pravosudov 2009; Roth et al. 2010b)). Second, environmental conditions may differ between native and introduced ranges (but see Prevosti et al. 1988 in the case of *D. subobscura*) and the level of cognitive ability may have changed due to selection, but this change might have been transient, and now be obscured by genetic assimilation (Lande 2015). While genetic assimilation is expected to happen if the new environmental conditions are predictable (Pigliucci et al. 2003; Crispo 2007), this latter hypothesis should account for the relatively low number of generations since introduction. It seems more likely that the first hypothesis – that the invasive individuals were pre‐adapted to the environmental conditions in their new range – accounts for our results. Invasion biologists in general may wish to precisely investigate the habitats and ecology of their biological model to locate putative ecological barriers that are relevant to evolution, rather than rely purely on geographical information, which is irrelevant to evolution (see Rey et al. 2012). For careful consideration, it should also be noted that the type of cognitive task investigated in our study is a basic learning task available to experimental manipulation in *D. subobscura* – far from the variety and complexity of behavioral tasks animals have to perform in nature (e.g., innovation in birds (Sol et al. 2002, 2005)). Our study hence tackled a modest, yet essential, part of possible cognitive evolution during the invasion of *D. subobscura* in South America.

One limitation of our study is that the relationship between fitness and our 24 h oviposition performance measurement may be weak in *D. subobscura*. Short‐term measurements of fecundity have not been well‐correlated with fitness in several species of Diptera, and measurements of lifetime fitness are to be preferred (e.g., *D. littoralis*, (Pekkala et al. 2011); *Musca domestica*, (Reed and Bryant 2004). Unfortunately, the correlation between fecundity and fitness has not been investigated directly in *D. subobscura*, even though this correlation has been assumed to be strong (e.g., Santos et al. 2010). However, *D. subobscura* is known to lay eggs and reach its peak fecundity early in life (start after 3 days and peak around 7 days; Maynard Smith 1958, Simões et al. 2008), and its fecundity remains relatively constant for at least 40 days (Maynard Smith 1958). Moreover, our investigation focused on the interaction between fecundity and learning skills that are measurable for no more than 1 day. Considering this time window, we chose to use fecundity of 7‐day‐old females as the best surrogate for lifetime reproductive success in *D. subobscura*. Other experimental designs that would investigate the interaction between fecundity and learning over the entire life span of *D. subobscura* individuals would not be feasible on our population scale (>12,000 egg‐laying females).

To our knowledge, this study is the first attempt to concomitantly investigate interpopulation variation in oviposition performance due to fecundity and behavioral plasticity in an insect species during the course of an invasion. Our results show no evolution of learning but increased fecundity, and no apparent trade‐off between these traits during *D. subobscura*'s expansion in South America. However, oviposition site choice is not the only trait that could trigger cognitive evolution during invasions. Introduced propagules may face greater cognitive challenges from unknown hazards such as new predators, pathogens, or competitors. Depending of the propagule size, locating potentials mates may also be crucial to the success of an invasion event. It would thus be essential to examine other aspects of behavior to thoroughly evaluate the importance of cognitive evolution during successful invasion events.

## Conflict of Interest

None declared.

## Supporting information


**Table S1.** Number of tests performed.
