# Supplementary material for: Introduced Drosophila subobscura populations perform better than native populations during an oviposition choice task due to increased fecundity but similar learning ability
Source: Ecol Evol. 2016 Feb 16;6(6):1725–36. doi: 10.1002/ece3.2015 (PMC4755011; doi:10.1002/ece3.2015)
Supplement: Supplementary file 1 — Table S1. Number of tests performed. [file ECE3-6-1725-s001.doc]

**Supporting Information**

Supplementary Table 1 : Number of tests performed.

Note : Each test correspond to a single group of 12 seven-day-old mated females.
